# The Fornix in Mild Cognitive Impairment and Alzheimer’s Disease

**DOI:** 10.3389/fnagi.2015.00001

**Published:** 2015-01-21

**Authors:** Milap A. Nowrangi, Paul B. Rosenberg

**Affiliations:** ^1^Department of Psychiatry and Behavioral Sciences, Johns Hopkins University School of Medicine, Baltimore, MD, USA

**Keywords:** Alzheimer’s, MCI, fornix, DTI, DBS

## Abstract

The fornix is an integral white matter bundle located in the medial diencephalon and is part of the limbic structures. It serves a vital role in memory functions and as such has become the subject of recent research emphasis in Alzheimer’s disease (AD) and mild cognitive impairment (MCI). As the characteristic pathological processes of AD progress, structural and functional changes to the medial temporal lobes and other regions become evident years before clinical symptoms are present. Though gray matter atrophy has been the most studied, degradation of white matter structures especially the fornix may precede these and has become detectable with use of diffusion tensor imaging (DTI) and other complimentary imaging techniques. Recent research utilizing DTI measurement of the fornix has shown good discriminability of diagnostic groups, particularly early and preclinical, as well as predictive power for incident MCI and AD. Stimulating and modulating fornix function by the way of DBS has been an exciting new area as pharmacological therapeutics has been slow to develop.

## Introduction

Alzheimer’s disease (AD) is the most common neurodegenerative condition in aging. AD is a growing public health problem that is projected to reach epidemic proportions if disease-modifying therapies are not found. The latest figures from the Alzheimer’s Association indicate that there are an estimated 5.3 million Americans living with AD. By 2050, an estimated 11–16 million people are expected to be diagnosed in the United States alone (Thies et al., 2013). Establishing the diagnosis of Mild Cognitive Impairment (MCI) and AD has evolved over the last 25 years with the most recent iteration of the National Institute on Aging and Alzheimer’s Association criteria (McKhann et al., 1984, 2011). These criteria place a new emphasis on the use of biomarkers of AD pathophysiology whereas the original criteria were based solely on the clinical evaluation. Although the use of biomarkers to establish diagnosis or track progression is considered a step forward in the field, there is a need for continued development of complementary technologies utilizing biological, physical, cognitive, and behavioral substrates.

Broadly, the initial pathologic changes in AD have been shown to involve the medial temporal lobes with the accumulation of neurofibrillary tangles and senile beta amyloid plaques (Bancher et al., 1993; Braak and Braak, 1995; Xu et al., 2000; Braak et al., 2006). This region is thought to mediate the retrieval and learning of semantic and episodic memory – the most common early cognitive deficits in AD (Aggleton and Brown, 1999; Behl et al., 2005; Levy and Chelune, 2007; Baldwin and Farias, 2009). The accumulation of tangles and plaques is associated with progressive atrophy of the cortical gray matter as loss of large pyramidal neurons (layers III and IV) advances, particularly in cortical association regions (Braak and Braak, 1995). In addition to this, several white matter (WM) abnormalities have been described and are thought to reflect axonal disintegration, rarefaction, oligodendrocytosis, and astrocytosis (Xu et al., 2001; Roher et al., 2002; Shahani et al., 2002). Neuroimaging techniques, particularly magnetic resonance imaging (MRI), is a robust method used to visualize and detect subtle changes in structure and function of the substructures within the medial temporal lobe and related regions as important and perhaps early markers of disease and progression.

Though most volumetric imaging studies focused on atrophy of the entorhinal cortex and hippocampus, close inspection of the limbic structures has revealed significant volume reductions in patients with sporadic and familial AD (Decarli, 2001; Cash et al., 2013). The fornix is one structure within the limbic system that is receiving increasing attention recently in part because of its ease of detectability using MRI scanning as well as robust associations to cognitive changes, diagnostic group discrimination, and susceptibility to therapeutic intervention (Aggleton and Brown, 1999; Thomas et al., 2011). Because of the increasing interest in this structure, this brief review will serve as an updated survey of the key research over the last 10 years involving the fornix with hopes for continued and increasing efforts to better understand the use of this structure in the diagnosis, progression, and treatment of AD.

## Fornix – Structure and Function

**Figure 1:**
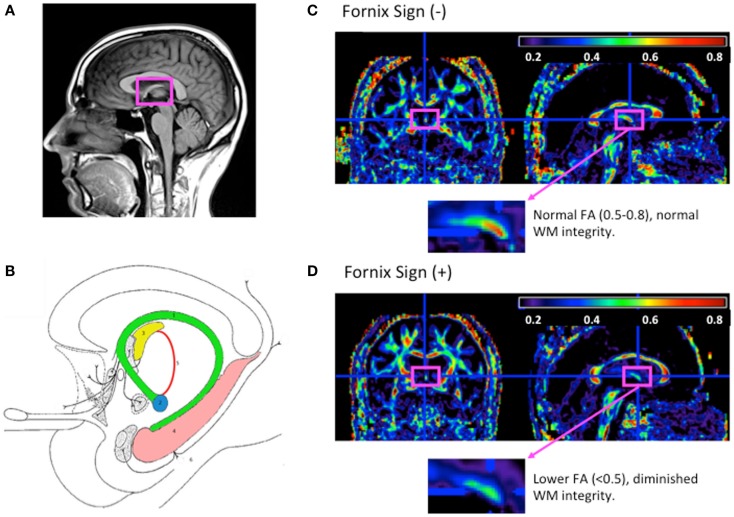
**The fornix anatomy and DTI fornix sign**. **(A)** Normal neuroanatomy highlighting limbic structures. **(B)** 1. Fornix; 2. mammilary body; 3. anterior thalamic nucleus; 4. hippocampus; 5. mammillothalamic tract; and 6. entorhinal cortex. Adapted from Nieuwenhuys (2008). **(C)** FA map of cognitively normal 80-year-old woman without fornix sign. **(D)** FA map of 80-year-old woman with Alzheimer’s disease with fornix sign. **(C,D)** FA maps shown with magnified view of the fornix (fuchsia rectangle), adapted from Oishi et al. (2011).

The fornix is a WM bundle belonging to the medial diencephalon, which also includes the hippocampus, mammillary bodies, and anterior and medial thalamus. Grossly, the fornix is found on the medial aspects of the cerebral hemispheres connecting the medial temporal lobes to the hypothalamus. It is formed at first from the output fibers of the hippocampus in the mesial temporal lobe, beneath the floor of the lateral ventricle. It courses along the curve of the corpus callosum forming its body. At the level of the foramen of Monro, the fornix divides and travels inferiorly and posteriorly in the lateral wall of the third ventricle to end in the hypothalamus and basal forebrain. Though the left and right fornices travel separately along their course from the hippocampus to the hypothalamus, they merge at the level of the forniceal body to form an important commissural tract (Nolte, 1993; Aggleton and Brown, 1999; Brewer, 2003; Patestas Ma, 2006). See Figures 1A,B.

The fornix is but one structure within the “temporal lobe memory system” also termed the Papez circuit described by James Papez in 1937 (Papez, 1995). The fornix has been demonstrated as the link between the hippocampus, mammillary bodies of the hypothalamus, and the anterior thalamic nuclei (via the mammilothalamic tract) (Nolte, 1993; Yamada et al., 1998; Aggleton and Brown, 1999; Patestas Ma, 2006). Efferent projections from the hippocampus to the medial diencephalon, particularly the anterior thalamic nuclei, are thought to be essential for normal hippocampal activity and are directed by the fornix, which then forms the major cholinergic output tract for the hippocampus (Cassel et al., 1997; Aggleton and Brown, 1999). Some of the earliest research involving lesion studies of this system asserted that damage to this axis resulted in anterograde amnesia, which occurs as a result of faulty encoding of episodic information (Aggleton and Brown, 1999). Specifically, damage to the hippocampus, fornix, and other diencephalic structures result in the inability to form declarative (semantic and episodic) memories. Although selective damage of the fornices has been shown to result in anterograde amnesia, the damage to other structures within the Papez circuit result in similar deficits emphasizes that the integrity of the circuit rather than individual substructure is important.

## Fornix as an Imaging Biomarker in MCI and AD

There has been increasing research in recent years on imaging the fornix as a biomarker for diagnosis, progression (or conversion), and cognition in MCI and AD. Some of the earliest research focused on fornix ultrastructure as a sign of MCI and AD. Much of this research employed MRI volumetric methods and showed atrophy (volume reduction) in many of the limbic lobe structures including the fornix. Early volumetric studies by Callen et al. (2001) showed that a number of structures within the Papez circuit (hippocampus, amygdala, anterior thalamus, hypothalamus, mammillary bodies, basal forebrain, septal area, fornix, orbitofrontal, and parahippocampal cortices) suffered significant atrophy with hippocampal and posterior cingulate regions being particularly affected. These early studies largely established complimentary findings to neuropathological observations of limbic atrophy in AD (Hopper and Vogel, 1976). The interest in establishing morphological changes in preclinical AD (MCI) as an early marker of disease was first undertaken by Copenhaver et al. (2006) who did not show significant atrophy of fornix or mammillary bodies in MCI as was initially hypothesized. The inability to show this difference was likely due to the relative insensitivity of volumetric methods used at the time even though imaging processes and analyses were improved upon compared to similar previous studies (Gale et al., 1993; Bilir et al., 1998; Kuzniecky et al., 1999; Callen et al., 2001). As a result, most recent research in the area has focused on fornix microstructure as the measurement of WM integrity. This renewed direction in imaging research of the fornix was based on the hypothesis that the earliest morphological changes occurred within medial temporal lobe gray matter, specifically the hippocampus and entorhinal structures with secondary effect to the efferent outflow tract through the fornix and mammillary bodies (Braak and Braak, 1995; Cassel et al., 1997; Aggleton and Brown, 1999). Needless to say, the underlying pathological processes affect an interconnected network of regions rather than single regions.

Diffusion tensor imaging has been the most commonly used *in vivo* MRI technique in studying the WM architecture of the fornix. Diffusion tensor imaging (DTI) is an indirect method of measuring WM integrity when conventional MRI lacks the contrast to delineate WM fiber tract organization (Beaulieu et al., 1996; Mori et al., 1999; Basser and Jones, 2002). Fractional anisotropy (FA) and mean diffusivity (MD) are two DTI measures used to quantify the integrity of WM microstructure by measuring the relative random motion of water in cerebral tissue. These measures are scalar values where higher values of FA and lower values of MD are thought to represent normal tissue cytoarchitecture where the random motion of water along a healthy axon, for example, is tightly constrained and restricted to movement along one direction (anisotropic). Two additional measures, axial diffusivity (DA) and radial diffusivity (DR) are associated with secondary degeneration of axons and breakdown of myelin and may provide additional detail into WM fiber integrity (Pierpaoli et al., 1996; Song et al., 2003, 2004).

Relating DTI measurement with other biomarkers that are considered more direct ways of detecting AD pathology has shown good correlation and has been a means of test validation. PET amyloid imaging is a well-established method of directly measuring AD pathology, β-amyloid (Aβ) deposition *in vivo*, but clinical application has been limited by convenience of use, invasiveness, and the half-life of radiotracers. When compared with DTI, several studies have shown positive correlation. A study by Chao et al. (2013) showed Aβ deposition as measured by Pittsburgh compound B (PiB) PET imaging was associated with reduced WM integrity (lower FA) in the fornix and splenium of the corpus callosum in subjects who ranged from normal to MCI. Similarly, Racine et al. (2014) showed a positive correlation between Aβ+ subjects and those with lower FA in the corpus callosum, hippocampal cingulum, and lateral fornix. Comparison with CSF markers of AD pathology Aβ_42_ and p-Tau_181_ has also been an important area of research. A study by Gold et al. (2014) showed a positive correlation between these CSF markers and WM integrity (FA) in the fornix, a relationship that persisted after controlling for hippocampal and fornix volume in a cohort of 20 cognitively normal older adults. Lower FA in the fornix was also associated with reduced performance on several cognitive tests including a Digit Symbol Test. This study is particularly important because CSF changes are thought to precede neuroimaging alterations by several years (Jack et al., 2010). These studies strongly suggest that microstructural measurements are related to AD pathology.

As the interest in the field of identifying early markers of AD continued to grow, several studies aimed to identify WM changes in preclinical AD. One of the earlier studies by Ringman et al. (2007) found decreased FA in the columns of the fornix were associated (linear regression) with hippocampal atrophy (*p* = 0.023), WM volume (*p* = 0.002), and mutation status (*p* = 0.032) in asymptomatic familial AD. They concluded that decreased FA in the forniceal columns is a robust finding in early familial AD and may be a biomarker of early disease in sporadic AD. Since this important study, there have been a number of more recent DTI studies showing similar associations between the WM tracts of the fornix and earlier preclinical stage. Cross-sectional DTI analysis by Mielke et al. (2009) and longitudinal analysis of the same cohort by Nowrangi et al. (2013) showed significant differences between normal participants and those with MCI and AD in the fornix, anterior cingulum, and splenium cross-sectionally and longitudinally (*p* < 0.01). Decreases in FA cross-sectionally and increases in MD longitudinally were observed in the fornix and were thought to indicate early markers of disease and longitudinal disease progression. Studies by Huang et al. (2012) and Boespflug et al. (2013) offered full DTI characterization (FA, MD, DA, and DR) of region of interest and atlas-based approaches to WM analysis respectively in MCI and AD. Both studies showed selective disruption of WM integrity in limbic structures including the fornix with DR being most sensitive to changes in the fornix. These studies further highlight a robust association between loss of integrity within the limbic structures and decrease in cognitive performance as illustrated by neuropsychological tests of episodic memory. Taken together, these studies suggest that the limbic WM tracts are preferentially affected in the early stages of cognitive dysfunction and that microstructural degradation of the fornix may precede atrophy of the hippocampus as detected by MRI.

Identifying preclinical signs of AD have been complemented by other research identifying individuals who would go on to progress to AD or convert from MCI to AD. One of the first studies observing this was by Oishi et al. (2011) who identified the fornix on an FA-scaled DTI map. FA reductions in this region predicted conversion form normal to amnestic MCI (aMCI) with a specificity of 1.0 and sensitivity of 0.67 and conversion from aMCI to AD with a specificity of 0.94 and sensitivity of 0.83. This finding was termed “the fornix sign” and has shown promise as an early predictive imaging sign of AD (See Figures 1C,D). Two other studies similarly emphasized the use of fornix microstructure as a predictor of conversion. One study by Van Bruggen et al. (2012) retrospectively used DTI analysis to differentiate between a subgroup of aMCI who converted to AD and a subgroup that did not. They found that FA, DR, and DA changes within the fornix, cingulum, and the corpus callosum were able to significantly discriminate between the two diagnostic groups and therefore could be thought of as a predictor for conversion between MCI and AD. Another study by Douaud et al. (2013) compared two groups of MCI patients; one that converted to AD no earlier than from baseline scanning and another that remained stable without progression of symptoms for at least 3 years. Comparing both volumetric and microstructural variables including DTI anisotropy and diffusivity, they found significant group differences in the body of the fornix, left fimbria, and the superior longitudinal fasciculus. These studies emphasize the importance of microstructural integrity of the limbic lobe and specifically the fornix in predicting the progression/conversion from preclinical to clinical AD.

Recently, the field has been quite interested in identifying cognitively normal individuals who might be at increased risk for developing AD. The well-known and well-regarded hypothesis by Jack et al. and others that imaging changes are evident before clinical symptoms arise and that the pathological changes in the brain begin even decades before those has prompted increased interest in identifying these individuals (Morris et al., 1996; Bennett et al., 2006; Jack et al., 2010, 2013). In line with previous hypotheses that microstructural change precedes gross atrophic changes, there has been increasing work in using DTI and complimentary imaging methods to examine this. One study by Fletcher et al. (2013) identified the fornix as a region of microstructural change in cognitively normal elders. They found that the fornix body volume and DA were highly significant predictors of cognitive decline from normal cognition [*r * = =0.47 (0.24–0.89), *p* = 0.02, and *r * = =1.25 (1.02–1.46), *p* = 0.005, respectively]. This is one of the first studies establishing fornix degeneration as a predictor of incipient cognitive decline among healthy elderly individuals. Two studies conducted by Zhuang et al. (2012, 2013) demonstrated that WM changes in the fornix were evident in preclinical AD. One study (Zhuang et al., 2013) showed that when compared to cognitively normal subjects and those with late MCI, early aMCI subjects had lower WM integrity in the fornix. In a second study (Zhuang et al., 2012), the same group showed that when compared to normal subjects who remained stable over 2 years, those who converted to aMCI had substantial reductions in WM integrity in the fornix, parahippocampal gyrus WM, and cingulum while gray matter structures remained relatively intact. FA was found to be a predictor of conversion from cognitively normal to aMCI. Taken together, these studies emphasize the continued importance of detecting earlier preclinical forms of AD even in normal subjects in order to identify patients who might benefit from interventional approaches. Table 1 summarizes the key studies described above.

**Table 1:** **Summary of key biomarker research of the fornix in cognitively normal (NC), MCI, and AD**.

Study	Cohort	Regions of interest and imaging findings	Conclusion
Ringman et al. (2007)	2 AD	FA for mean whole brain WM, forniceal columns, bilateral perforant pathways, left orbitofrontal lobe were decreased relative to non-carriers	FA is reduced in the fornix in persons carrying mutations for AD prior to symptoms of dementia and is a better predictor of mutation status than other regions
	21 at-risk AD	
Mielke et al. (2009)	25 NC	Lower FA in fornix, anterior cingulum bundle, splenium of the corpus collosum in AD vs. NC and AD vs. MCI	FA is decreased in specific fiber tracts including the fornix in NC, MCI, and AD and may be an indicator of progression over 3 months
	25 MCI	
	25 AD	
Nowrangi et al. (2013)	25 NC	Higher MD and lower FA in the fornix and splenium in AD vs. MCI or NC of 12 months	Higher MD in the fornix longitudinally was a better indicator of change than FA and may be an early indicator of progression
	25 MCI	
	25 AD	
Huang et al. (2012)	24 NC	FA, MD, DR, DA of limbic, commissural, and association tracts are differentially associated with diagnostic group. Comparison between aMCI and NC show differences only in limbic structures	WM disruption of limbic tract structures is caused by neuronal damage in aMCI and indicates a progression pattern between WM tracts
	11 aMCI	
	26 AD	
Boespflug et al. (2013)	18 MCI	Lower DR, higher FA, and lower MD in the fornix associated with better paired associate learning	Disparate pathology of temporal stems and fornix WM is associated with early memory impairment in MCI
Oishi et al. (2011)	25 NC	Fornix sign differentiated AD vs. NC and predicted conversion from NC to aMCI and from aMCI to AD	The fornix sign may be a predictive biomarker sign of AD
	24 aMCI	
	23 AD	
Van Bruggen et al. (2012)	15 NC	Significant differences in FA and DR in the fornix, corpus callosum, and cingulum in MCI who remained stable vs. converters	DTI changes in MCI converters vs. those who remained stable may be an early indicator of progression to AD
	17 MCI (8 stable, 9 converters)	
	15 AD	
Douaud et al. (2013)	13 aMCI (converters)	Significant group differences in volume and microstructure of left hippocampus, body of the fornix, left fibria, and superior longitudinal fasciculus	Microstructural changes in left hippocampus using DTI showed most substantial differences between two diagnostic groups and was best predictor of future progression to AD
	22 aMCI (stable)	
Fletcher et al. (2013)	102 NC	Fornix body volume and DA were highly significant predictors of cognitive decline from normal cognition	Fornix degeneration in NC may be a predictor of incipient cognitive decline among healthy elderly individuals
Zhuang et al. (2012)	173 NC (stable)	aMCI converters had substantial reduction in FA in precuneus, parahippocampal cingulum and gyrus, and fornix while gray matter structures intact	Microstructural WM changes are present in NC in the pre-aMCI stage and may be an imaging marker of early AD-related brain changes
	20 aMCI (converted)	
Zhuang et al. (2013)	155 NC	Late aMCI had lower WM integrity in the fornix, parahippocampal cingulum, and uncinate fasciculus, early aMCI showed white matter damage in fornix	Limbic WM tracts preferentially affected in early stages of cognitive dysfunction particularly in the fornix, which may precede hippocampal atrophy
	27 “late” aMCI	
	39 “early” aMCI	

## Fornix and Clinical Treatment in AD

One of the most recent and exciting avenues of research has been directed at enhancing cognition by stimulating or modulating fornix function. Deep Brain Stimulation (DBS) has been thought of for some time now as having significant effect on mood and anxiety disorders such as major depression, obsessive compulsive disorder as well as Gilles de Tourette’s syndrome, and other conditions. Similarly, it has been hypothesized that DBS may also play a role in enhancing memory functions. One of the first studies in this area by Hamani et al. (2008) used DBS to stimulate the fornix/hypothalamus area in a man with morbid obesity. In a case observation report, they also described that the procedure generated detailed autobiographical memory. In AD, this same concept has been hypothesized as being a potential therapeutic intervention for the treatment of amnesia and has just started being seriously studied. The first study of its kind, by Laxton et al. (2010), performed DBS stimulation of the fornix and hypothalamus in six AD patients. Though this was largely a safety study, the authors found that DBS triggered neural activity (increased metabolism) in some patients as seen through PET imaging (Laxton et al., 2010).

Later *in vivo* studies of DBS in patients with AD have been few and largely with relatively low *N*. Since 1985, there have been just two DBS studies targeting the stimulation of the fornix in patients with AD (Laxton et al., 2010; Fontaine et al., 2013). Found to over-all be safe, the clinical response has been rather modest with MMSE scores stabilizing rather than improving. However, reversal of AD-related hypometabolism has been observed in temporo-parietal-occipital regions as well as increased connectivity between similar regions representing distributed cognitive networks (Smith et al., 2012). Given the lack of success thus far in developing pharmacological therapies for AD and the slow pace of further developments, such novel treatments as DBS is a promising avenue for therapy in AD. Currently, the Advance DBS clinical trial has recently completed recruitment of 42 participants with mild AD. Half of the study participants will have the stimulators turned on at the start of the study and the other half 12 months later. All participants will be followed for 18 months to track progression of their cognitive impairment. More information of this study can be found at www.clinicaltrials.gov.

## Conclusion

There is a small but growing body of research directed toward the structure and function of the fornix in MCI and AD. Because of its important role within the “temporal lobe memory system,” understanding the microstructural integrity of the fornix in MCI and AD has become an important area of focus in the field as there is a growing priority for identifying individuals early in the disease process and those who are at increased risk for progression or conversion. Recent studies have shown DTI is able to indirectly measure microstructural changes of the fornix that may precede gross atrophic changes of gray matter structures such as the hippocampus and entorhinal cortex. These studies demonstrate that DTI measurements of the fornix can significantly discriminate between cognitively normal, MCI, and AD groups thereby serving as a robust biomarker of disease. Moreover, fornix measurements have been shown to be a reliable measure of conversion from preclinical disease to AD. As pharmacological interventions continue to develop, studies of stimulating the fornix through DBS to alleviate or even reverse cognitive impairment has shown some early promise in stabilizing cognitive decline and increasing neural network activity.

## Conflict of Interest Statement

The authors declare that the research was conducted in the absence of any commercial or financial relationships that could be construed as a potential conflict of interest.
